# A Novel Mouse Model of a Patient Mucolipidosis II Mutation Recapitulates Disease Pathology*

**DOI:** 10.1074/jbc.M114.586156

**Published:** 2014-08-08

**Authors:** Leigh Paton, Emmanuelle Bitoun, Janet Kenyon, David A. Priestman, Peter L. Oliver, Benjamin Edwards, Frances M. Platt, Kay E. Davies

**Affiliations:** From the ‡ Medical Research Council Functional Genomics Unit, Department of Physiology, Anatomy, and Genetics, University of Oxford, South Parks Road, Oxford OX1 3PT, United Kingdom and; the § Department of Pharmacology, University of Oxford, Mansfield Road, Oxford OX1 3QT, United Kingdom

**Keywords:** Ataxia, Drug Action, Lysosomal Storage Disease, Mouse Genetics, Neurodegeneration, Mucolipidosis II, NPC2

## Abstract

Mucolipidosis II (MLII) is a lysosomal storage disorder caused by loss of *N*-acetylglucosamine-1-phosphotransferase, which tags lysosomal enzymes with a mannose 6-phosphate marker for transport to the lysosome. In MLII, the loss of this marker leads to deficiency of multiple enzymes and non-enzymatic proteins in the lysosome, leading to the storage of multiple substrates. Here we present a novel mouse model of MLII homozygous for a patient mutation in the *GNPTAB* gene. Whereas the current gene knock-out mouse model of MLII lacks some of the characteristic features of the human disease, our novel mouse model more fully recapitulates the human pathology, showing growth retardation, skeletal and facial abnormalities, increased circulating lysosomal enzymatic activities, intracellular lysosomal storage, and reduced life span. Importantly, MLII behavioral deficits are characterized for the first time, including impaired motor function and psychomotor retardation. Histological analysis of the brain revealed progressive neurodegeneration in the cerebellum with severe Purkinje cell loss as the underlying cause of the ataxic gait. In addition, based on the loss of Npc2 (Niemann-Pick type C 2) protein expression in the brain, the mice were treated with 2-hydroxypropyl-β-cyclodextrin, a drug previously reported to rescue Purkinje cell death in a mouse model of Niemann-Pick type C disease. No improvement in brain pathology was observed. This indicates that cerebellar degeneration is not primarily triggered by loss of *Npc2* function. This study emphasizes the value of modeling MLII patient mutations to generate clinically relevant mouse mutants to elucidate the pathogenic molecular pathways of MLII and address their amenability to therapy.

## Teaser

**Background:** Mucolipidosis II is a severe lysosomal storage disorder, fatal in childhood and lacking drug treatments.

**Results:** This novel mouse model of a mucolipidosis II patient mutation recapitulates the human pathology.

**Conclusion:** Mouse models based on patient mutations are more valuable to study mucolipidosis II than a knock-out of the gene.

**Significance:** This novel mouse model will be useful for future drug development.

## Introduction

The rare autosomal recessive lysosomal storage disorder MLII^5^ (originally called I-cell disease (1)) presents with delayed motor milestones and cognitive impairments, severe skeletal abnormalities, coarse facial features, thickened skin, and early death in the first decade of life due to cardiac and pulmonary failure (2, 3). The disease is caused by the loss of multiple hydrolases in the lysosome due to a defect in their targeting to lysosomes. Waste material in the cell is targeted to the lysosome by the endocytic or autophagic pathways. Here lysosomal enzymes degrade and recycle this waste material. Thus, this mechanism is important for the maintenance of cells and tissues (4). The majority of acid hydrolases are targeted for transport to lysosomes via the presence of a surface mannose 6-phosphate (M6P) marker that is recognized by its cognate receptor (5–9). The protein responsible for the synthesis of the M6P marker is dysfunctional in MLII.

Both MLII-causing *GNPTAB* homozygous and compound heterozygous nonsense and frameshift mutations, leading to premature termination codons, have been described. These result in the total loss of the hexameric (α_2_β_2_γ_2_) GlcNAc-1-phosphotransferase (GNPTA) enzyme activity (10–12). GNPTA catalyzes the first step in the synthesis of the M6P marker. Its subunits are encoded by the genes *GNPTAB* and *GNPTG. GNPTAB* encodes an initially enzymatic inactive transmembrane precursor protein (13, 14), which is cleaved by the site-1 protease to release catalytically active α- and β-subunits (15). *GNPTG* encodes the soluble γ-subunits of the GNPTA complex and has been shown to facilitate the recognition process (16).

Loss of GNPTA function leads to missorting and hypersecretion of lysosomal enzymes into the circulation, making them detectable in the blood sera of MLII patients (17). Due to the lack of hydrolases in the lysosomes, their substrates accumulate, leading to lysosomal “storage.” Animal models of MLII have been described with the feline model of MLII (18) recapitulating the human disease most closely, including coarse facial features, behavioral dullness, ataxia, and reduced life span (18, 19). *Gnptab*-depleted zebrafish embryos have been engineered using morpholinos and showed skeletal abnormalities, craniofacial defects, and reduced motility. In addition, developmental studies were more accessible with this model, and changes in the expression pattern of chondrogenic factors were shown (20, 21). A *Gnptab* gene trap mouse model has also been described and is characterized by impaired growth, retinal degeneration, lesions in secretory epithelial cells of exocrine glands, and elevated levels of serum acid hydrolases (22, 23). This mutant presented with a relatively normal life span and did not develop characteristic disease features, such as skeletal and facial abnormalities. Here, we describe a novel mouse model, which was recovered from an *N*-ethyl-*N*-nitrosourea screen, termed Nymphe (*nym*), which carries the previously reported patient mutation Y888X (12). Importantly, the Nymphe mouse (*nym/nym*) recapitulates the major features of the human disease and, for the first time, enables a detailed behavioral characterization of the motor dysfunction and psychomotor retardation. Histological analysis of the brain revealed progressive neurodegeneration of Purkinje neurons in the cerebellum, probably the underlying cause of ataxia. Also, there was complete loss of Npc2 protein expression. Purkinje cell pathology in the Nymphe mouse was treated with 2-hydroxypropyl-β-cyclodextrin, a drug previously reported to delay Purkinje cell loss in a mouse model of NPC disease. This approach did not rescue Purkinje cell loss, indicating that the loss of Npc2 expression in the Nymphe mouse brain is not the primary molecular mechanism triggering Purkinje cell degeneration.

## EXPERIMENTAL PROCEDURES

### 

#### 

##### Animals

Animal work was approved by the University of Oxford Ethics Panel and was carried out in accordance with United Kingdom Home Office regulations. The *nym*/*nym* mouse was identified from a phenotype-driven screen of the progeny from Balb/cAnNHsd *N*-ethyl-*N*-nitrosourea mutagenized mice crossed to female C3H/HeNHsd engineered at MRC Harwell. The colony was maintained by back-crossing against C3H/HeNHsd.

##### Genetic Mapping and Mutation Detection

Mutant mice were initially screened for genome-wide SNP markers between the parental C3H/HeH and BALB/c (*N*-ethyl-*N*-nitrosourea-treated) strains, followed by mapping using additional microsatellite markers to an interval between D10Mit42 and D10Mit178. Further fine mapping was carried out to reduce the critical subchromosomal region to 6 Mb between SNPs 86559223 and 92592980 (NCBI build 37). The *nym* mutation was identified by PCR and direct sequencing of all genes present within this interval, including the coding regions and exon-intron boundaries (primer sequences are available upon request). Analysis of mutant mice was conducted after a minimum of 15 back-crosses, ensuring only the subchromosomal region being contained within the wild-type background. Genotyping for the presence of the *nym* mutation was carried out using the primers *nym* forward (5′-GGAGACGGTGACATACAAAAATCT-3′) and *nym* reverse (5′-CACTGGATGCTCTAAGGAAGATAT-3′) and subsequent digest with MseI because this can cleave when the mutation is present.

##### RNA Extraction and RT-PCR

Whole brain was dissected from 3-month-old wild type and *nym*/*nym* mice. RNA was extracted using the RNeasy kit (Qiagen). Total RNA was reverse transcribed using Expand reverse transcriptase (Roche Applied Science). Total cDNA and genomic DNA were subjected to semiquantitative analysis. Cycling conditions were as follows: Gnptab, 1 μg of cDNA using 29 cycles; 18 S, 1 μg of cDNA using 16 cycles. Primers used were *Gnptab* forward (5′-GGCCTCAGAGTCAGAAAG-3′), *Gnptab* reverse (5′-CAACGCAAGCATAAAACAGC-3′), 18 S forward (5′-GCGGCTTGGTGACTCTAGAT-3′), and 18 S reverse (5′-CCCTCTCCGGAATCGAAC-3′). Samples were run in duplicates, and the sample loading was normalized by using the 18 S loading control. Blots were analyzed using ImageJ, and bands were quantified.

##### Plasmid Construction

The full-length cDNA sequence of mouse Gnptab (NM_001004164) was subcloned into pcDNA3 (Invitrogen) in frame with a C-terminal c-Myc tag for expression in mammalian cells. Mutant versions of this construct containing the *nym* mutation were engineered by QuikChange site-directed mutagenesis (Agilent Technologies) according to the manufacturer's instructions (primer sequences available upon request).

##### Cell Culture, Transfection, and Immunofluorescence

HEK 293 cells and mouse embryonic fibroblasts (MEFs) were cultured in DMEM supplemented with l-glutamine, 10% FBS, and 1% penicillin/streptomycin at 37 °C, 5% CO_2_. MEFs were isolated at day 12.5 after terminated mating. Cells were seeded onto poly-l-lysine-coated glass coverslips. pCDNA3-*Gnptab* wild-type and *nym*/*nym* mutant constructs were transfected into HEK 293 cells using Fugene 6 (Roche Applied Science). pEGFP-N1 (Clontech) was used to control for transfection efficiency. For immunocytochemistry, cells were fixed, blocked, and stained for 1 h at room temperature each with primary (Myc (1:200 dilution) from Sigma; GM130 (1:250) from Abcam; protein-disulfide isomerase (1:150) from Abcam) and Alexa Fluor-conjugated secondary antibodies (1:400; Invitrogen). Slides were imaged under a phase-contrast microscope (Leica), and images were captured using the Axiovision software (Axiocam).

##### Western Blotting

Tissue extracts were prepared in 10 mm Tris-HCl, pH 8, 10 mm NaCl, 1 mm EDTA, pH 8, 1% Triton X-100, and protease inhibitors (Roche Applied Science). Protein concentration of the lysates was determined by a BCA assay (Pierce). After primary antibody (anti-β-tubulin-1 (1:1000) was obtained from Sigma and anti-NPC2 (H-125) (1:100) from Santa Cruz Biotechnology, Inc.) and peroxidase-conjugated secondary antibody (1:5000) incubation (Invitrogen), blots were developed with the ECL kit (GE Healthcare). Band intensity relative to internal controls was analyzed using ImageJ software.

##### Immunohistochemistry and Histology

For immunohistochemical analysis, the trachea and pancreas were dissected, fixed overnight in 4% paraformaldehyde, and cryoprotected in 30% sucrose, and tissue was embedded in OCT and sectioned. The tissue sections were stained with H&E for histopathological examination. To investigate CNS pathology, mice were trascardially perfused with 4% paraformaldehyde, and brains were dissected and postfixed overnight and cryoprotected in sucrose before embedding in OCT. Slides were blocked for 1 h and incubated overnight at 4 °C with primary antibodies (anti-D28K calbindin (1:10,000) from Swant; anti-GFAP (1:400) from Sigma). Primary antibody staining was visualized using the Vectastain ABC Elite kit (Vector Labs) or Alexa Fluor 488 secondary antibodies (Invitrogen) for immunofluorescence. For luxol fast blue staining, fresh frozen brain sections were incubated at 56 °C overnight in 0.1% luxol fast blue solution (Solvent Blue 38, Sigma). Excess stain was rinsed off with 95% ethyl alcohol followed by distilled water. Slides were differentiated in 0.05% lithium carbonate solution for 2 min, followed by 70% ethyl alcohol for 1 min, and rinsed in distilled water. This step was repeated three times until the gray matter was clear, and the white matter was sharply defined. Periodic acid-Schiff staining was carried out using a periodic acid-Schiff kit (Sigma) as instructed by the manufacturer. Filipin complex (Sigma) was used at a working concentration of 10 μg/ml. Brain sections were incubated for 3 h at room temperature in the dark. Slides were imaged as described above.

##### Lysosomal Enzyme Assay

Blood sera were collected from adult wild-type (*n* = 8), *nym*/+ (*n* = 8), and *nym/nym* mice (*n* = 6) at 12 weeks of age by severing the jugular vein after CO_2_ narcosis. Blood serum was separated by centrifugation and stored at −20 °C until assayed. Activities of β-hexosaminidase were assayed with 5 mm 4-nitrophenyl *N*-acetyl-β-d-glucosaminide (Sigma), β-galactosidase with 5 mm *p*-nitrophenyl-β-d-galactopyranoside (Sigma), β-glucuronidase with 5 mm 4-nitrophenyl β-d-glucuronide (BioChemika), α-mannosidase with 5 mm 4-nitrophenyl α-d-mannopyranoside (Sigma), β-mannosidase with 5 mm 4- nitrophenyl β-d-mannopyranoside (Sigma), α-galactosidase with 5 mm 4-methylumbelliferyl-α-d-galactopyranoside (Sigma), and β-glucocerebrosidase with 5 mm 4-methylumbelliferyl-β-glucoside. Blood sera and brain lysate was incubated with 5 mm substrate at 37 °C for 1 h. Reactions were stopped by the addition of 0.1 m glycine NaOH solution (pH 10.3), and the fluorescence was read at 399 nm. Activities were expressed as nmol of substrate cleaved/mg of protein/h. The specific activities of the wild-type were set to 1, and -fold changes of *nym/nym* are expressed as ratio to the wild-type.

##### Behavioral Testing

Behavioral testing was carried out on mice at 3, 7, and 11 months of age.

##### Rotarod

A commercial rotarod device was used (Accelerating model, Ugo Basile, Biological Research Apparatus, Varese, Italy) consisting of a grooved plastic beam 5 cm in diameter. Mice were placed on the beam (revolving at the default 5 rpm), and after 1 min, the rod speed was gradually accelerated to a maximum of 30 rpm over 4 min by electronic control of the motor. Latency to fall in each trial was recorded.

##### Inverted Screen

A 90-cm^2^ screen of wire mesh consisting of 12 mm^2^ of 1-mm diameter wire surrounded by a 10-cm-deep wooden frame was used. The mouse was placed in the center of the wire mesh screen and immediately rotated. The screen was maintained 27 cm above a padded surface. Latency of how long the mice remained upside down on the screen was measured, with a maximum score of 180 s.

##### Spontaneous Alternation Y-maze

Maze testing was carried out as described previously (25).

##### Catwalk Automated Quantitative Gait Analysis

Abnormalities in gait were assessed using the Noldus Catwalk gait analysis system (26). Mice were allowed to freely transverse the glass walkway while the video camera recorded the paw contact points. The Catwalk software then assigned identities to the respective paw prints recorded, generating a wide range of parameters. The regularity index acts as a measure of generalized coordination (27) by computing whether the mouse footfalls fall within regular step patterns. Gait regularity tests how consistently the mouse takes “normal strides” compared with “abnormal strides.” The base of support is the average width between either the front paws or the hind paws.

## RESULTS

### 

#### 

##### Gnptab Is the Gene Mutated in the Nymphe Mouse

**FIGURE 1.**
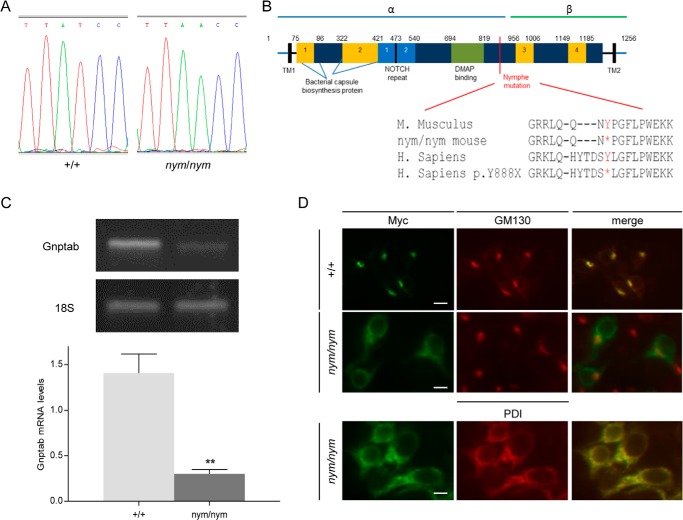
***Gnptab* is the gene mutated in *nym* mutant.** *A*, sequencing of the *Gnptab* locus identified a single nucleotide change resulting in a coding change from a tyrosine residue (TAT) to a premature stop codon (TAA) in the *nym* (*nym/nym*) mouse. *B*, schematic of human GNPTA α/β subunits presenting the location of the *nym* mutation and its conservation from mice to humans (indicated by an *asterisk* in *nym* mutants and for the human (*H. sapiens*) mutation Y888X). The precursor protein is cleaved between Lys-928 and Asp-929 by the site 1 protease to produce two catalytically active α and β subunits. *TM*, transmembrane domain; *aa*, amino acid. This figure is adapted from Ref. 59. *C*, semiquantitative RT-PCR analysis (*n* = 4) reveals that the mRNA is reduced by 75%. Results are expressed as relative levels after normalization for the internal control 18 S. *D*, intracellular localization of wild-type and mutant Gnpta in HEK 293 cells. Cells were fixed and stained with monoclonal antibodies against the Myc tag (*green*), the *cis*-Golgi marker protein GM130 (*red*), or the ER marker protein, protein-disulfide isomerase (*PDI*; *red*). In *merged images*, *yellow* indicates colocalization. *Scale bars*, 15 μm. Values are expressed as mean S.E. (*error bars*) (*n* = 8; *, *p* < 0.05; **, *p* < 0.01).

We isolated the recessive *nym* mouse from a large scale chemical mutagenesis screen that we are exploiting to uncover novel genes and pathways essential for the maintenance of nervous system function. This mutant was selected based on its smaller size and ataxic gait. Haplotype analysis localized the *nym* mutation to a genetic interval of 6 Mb on mouse chromosome 10, between markers *D10Mit42* and *D10Mit178*. PCR and direct sequencing of all 28 protein-coding genes within the interval including exonic regions and exon-intron boundaries revealed a single non-synonymous homozygote point mutation in exon 13 of the *Gnptab* gene. This mutation introduces a T to A substitution at nucleotide 2601 of the cDNA sequence (T2601→A) (Fig. 1*A*) that changes the tyrosine into a premature stop codon at position 867 of the protein sequence (Y867X) within an evolutionarily conserved spacer region 40 residues upstream of the cleavage signal between the α- and β-subunits (Fig. 1*B*). This is predicted to result in the production of a slightly truncated α-subunit (∼95% of full length) and a complete lack of the β-subunit. In agreement with the loss of a functional Gnpt enzyme, the counterpart *nym* mutation in the human protein sequence (Y888X) has, in fact, recently been identified in an MLII patient (12). Consistent with nonsense-mediated decay, *Gnptab* transcript levels were reduced by 75% in *nym* mice compared with wild type (Fig. 1*C*). The endoplasmic reticulum (ER) export signal is deleted in the *nym* mutation, and the truncated protein would be expected to remain in the ER; thus, the cellular localization of the mutant protein was investigated. The cytoplasmic domain of the β-subunit contains the (R/K)X(R/K)-type ER export signal and a C-terminal valine, which are both required for cargo-receptor-mediated packaging into COPII vesicles and trafficking of the GNPT precursor protein to the Golgi, where it gets cleaved (28, 29). Subcellular localization of the *nym* Gnpta was monitored in transfected HEK 293 cells. As expected, the wild-type Gnpta protein colocalized with the *cis*-Golgi marker GM130 (Fig. 1*D*, *top*), whereas the *nym* Gnpta protein did not (Fig. 1*D*, *middle*). The *nym* Gnpta protein remained trapped in the ER, as demonstrated by colocalization with protein-disulfide isomerase (Fig. 1*D*, *bottom*, *PDI*), an ER marker. These results confirm that the *nym* mutation indeed leads to a dysfunctional Gnpta enzyme and imply that the *nym* mouse is a novel model of MLII. These findings suggest that truncation mutations present in GNPTAB inhibit ER exit (30).

##### Growth Retardation and Facial and Skeletal Abnormalities Are Part of the Pathological Features of MLII in the Nymphe Mutant

**FIGURE 2.**
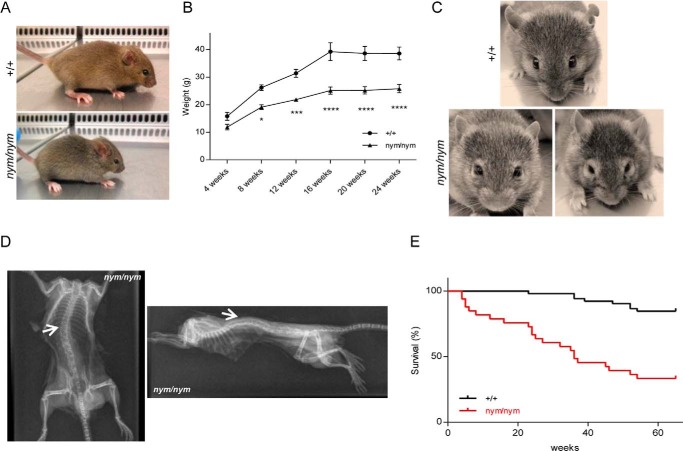
**Growth retardation, facial dysmorphism, and reduced life span.** *A*, 1-month-old *nym* (*nym/nym*) mice show reduced body size and skeletal abnormalities (*e.g.* facial dysmorphism and curvature of the spine) in comparison with wild-type (+/+) littermates. *B*, body weight progression of *nym* mice is reduced compared with wild-type littermates. *C*, facial phenotype progresses with age. Particularly evident is the thickening of the eyelids. *D*, whole body exotic dorso-ventral view of the 12-month-old *nym* mouse (*left*) and whole body exotic lateral view of the 12-month-old *nym* mouse (*right*). *White arrows*, abnormal curvature (*left*) and hunched back (*right*). *E*, Kaplan-Meier analysis of wild-type and *nym* mice (+/+, *n* = 52; *nym/nym*, *n* = 33). Values are expressed as mean ± S.E. (*error bars*) (*n* = 8; *, *p* < 0.05; **, *p* < 0.01; ***, *p* < 0.001; ****, *p* < 0.0001).

Prominent features of MLII are growth retardation and facial and skeletal abnormalities (12). Indeed, *nym* mutants remained significantly smaller than wild-type littermates (∼60%) throughout life (Fig. 2, *A* and *B*). Facial and skeletal abnormalities were evident from birth, including thickened eyelids and a flat profile with reduced nasal bridge (Fig. 2, *A* and *C*), as well as severe deformities that affect most prominently the back, in particular the spine, identified as kyphosis (Fig. 2*D*). In addition, *nym* mutants had an unusually stiff and thick skin. This presentation indeed resembles closely that of MLII patients with the same characteristic features. *nym* mice also displayed increased mortality compared with controls, and survival fell below 50% by 60 weeks of age (Fig. 2*E*).

**FIGURE 3.**
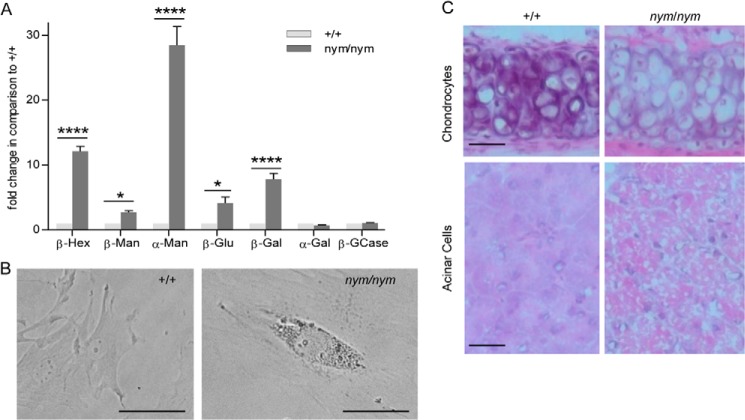
**Increased lysosomal enzyme activity in blood sera and inclusion bodies present in mouse fibroblasts, secretory and connective tissue.** *A*, the relative enzymatic activities of the lysosomal hydrolases β-Hexosaminidase (β-*Hex*), β-mannosidase (β-*Man*), α-mannosidase (α-*Man*), β-glucuronidase (β-*Glu*), β-galactosidase (β-*Gal*), α-galactosidase (α-*Gal*), and β-glucocerebrosidase (β-*GCase*) were measured in blood sera of 3-month-old wild-type (+/+) and *nym (nym/nym*) mice. The specific activities of the wild-type were set to 1. Values are expressed as mean ± S.E. (*error bars*) (*n* = 8; *, *p* < 0.05; **, *p* < 0.01; ***, *p* < 0.001; ****, *p* < 0.0001). *B*, light microscopy imaging of MEFs isolated from wild-type and *nym* embryos (E12.5). Accumulation of inclusion bodies present in the *nym* MEFs. *Scale bar*, 40 μm. *C*, *top*, the cytoplasm of hypertrophic chondrocytes is distended by microvacuoles in the *nym* mouse. These inclusions are aggregates of polysaccharides that increase in storage material with age. *Bottom*, marked disorganization of the pancreas in the *nym* mouse with tightly packed cells distended by large vacuoles. *Scale bar*, 50 μm.

Characteristic features of the pathology of MLII, used for the diagnosis of patients, are increased activities of lysosomal hydrolases found in the blood sera and intracellular accumulation of inclusion bodies (31). In the serum of *nym* mice, activities of the lysosomal hydrolases β-hexosaminidase, β-galactosidase, α-mannosidase, and β-mannosidase were increased by 2.5–30-fold compared with wild-type controls (Fig. 3*A*), which is consistent with the values reported in the *Gnptab* knock-out mouse and MLII patients (32). Lysosomal enzyme activities for blood sera in nmol/min/ml can be seen in Table 1. Fibroblasts, secretory organs, and connective tissue have been shown to be severely affected by inclusion bodies in MLII. Hence, we analyzed cytoplasmic inclusions by staining MEFs, pancreatic acinar cells as a sample for secretory tissue and chondrocytes in the cartilage of the trachea as a sample for connective tissue. The characteristic inclusion bodies (indicative of lysosomal storage) were clearly present in embryonic fibroblasts from *nym* mice (Fig. 3*B*). Chondrocytes in the cartilage of the trachea were enlarged, with the cytoplasm filled by inclusion bodies and abundant microvacuoles (Fig. 3*C*, *top*). In contrast, wild-type chondrocytes had low basic cytoplasm, containing a single clear vacuole. In addition *nym* chondrocytes were not affected by fixation-dependent shrinkage as much as wild-type chondrocytes (Fig. 3*C*, *top*). Pancreatic sections showed disorganization of tissue structure, including enlargement of acinar cells by the presence of large vacuoles containing faint granular material (Fig. 3*C*, *bottom*). Similar findings have previously been reported in the *Gnptab* gene trap mouse (22).

**TABLE 1:** **Lysosomal enzyme activities (nmol/min/ml) in blood sera of wild-type, heterozygote (*nym*/+), and *nym* (*nym/nym*) mouse**

Lysosomal enzyme	Wild type (+/+)	*nym*/+	*nym/nym*
β-Hexosaminidase (nmol/min/ml)	86.94 ± 7.32	144.02 ± 7.21	1094.69 ± 68.90
β-Mannosidase (nmol/min/ml)	10.49 ± 0.57	11.88 ± 1.76	29.07 ± 1.84
α-Mannosidase (nmol/min/ml)	50.30 ± 4.12	119.13 ± 7.23	1433.02 ± 144.98
β-Glucoronidase (nmol/min/ml)	6.96 ± 0.54	6.43 ± 1.06	28.88 ± 6.46
β-Galactosidase (nmol/min/ml)	8.13 ± 1.24	6.98 ± 0.93	63.58 ± 7.30
α-Galactosidase (nmol/min/ml)	2.12 ± 0.25	1.90 ± 0.13	2.20 ± 0.13
Glucocerebrosidase (pmol/min/ml)	3.31 ± 0.4	3.36 ± 0.45	3.47 ± 0.28

**FIGURE 4.**
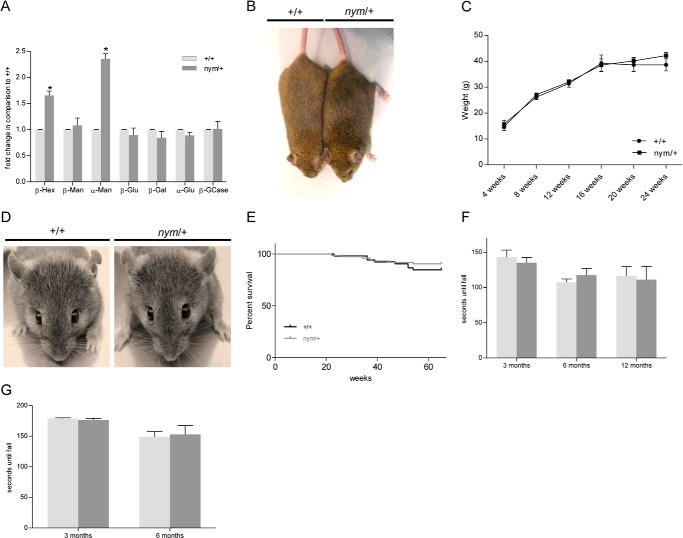
**The *nym* heterozygote mouse shows no obvious pathology except for an increase in enzymatic activity similar to carriers of mucolipidosis II.** *A*, the relative enzymatic activities of the lysosomal hydrolases β-Hexosaminidase (β-*Hex*), β-mannosidase (β-*Man*), α-mannosidase (α-*Man*), β-glucuronidase (β-*Glu*), β-galactosidase (β-*Gal*), α-Galactosidase (α-*Gal*), and β-glucocerebrosidase (β-*GCase*) were measured in blood sera of 3-month-old wild-type (+/+) and *nym*/+ mice. The specific activities of the wild type were set to 1. Three-month-old heterozygote (*nym*/+) mice are similar in size to the wild-type (+/+) littermates (*B*), and body weight progression of *nym*/+ mice is not significantly different from that of wild-type mice (*C*). *D*, facial phenotype of the *nym*/+ and wild-type mouse are no different. *E*, Kaplan-Meier analysis of wild-type and *nym*/+ mice (+/+, *n* = 52; *nym*/+, *n* = 82). *nym*/+ mice present with the same motor coordination on the rotarod (*F*) and muscle strength (*G*), which was measured on the inverted screen, as the wild-type littermate. Values are expressed as mean ± S.E. (*error bars*) (*n* = 8; *, *p* < 0.05; **, *p* < 0.01; ***, *p* < 0.001; ****, *p* < 0.0001).

Mating of *nym* mutants was only successful in 9% of breeding pairs, indicating infertility or incapacity of females to carry pups to term. Furthermore, *nym* mutants were born with a reduced frequency of 15% instead of the expected 25% Mendelian frequency, which implies reduced survival *in utero*. An increased frequency of *nym* males also presented with penile prolapse from 3 months of age. Despite a 1.7-fold increase in β-hexosaminidase and 2.4-fold increase in α-mannosidase (Fig. 4*A*) for the heterozygote (*nym*/+) mice in comparison with the wild-type control, no overt phenotype was displayed in the *nym*/+ mice. The *nym*/+ mice were indistinguishable from wild-type controls in fertility, size, body weight progression (Fig. 4, *B* and *C*), facial features (Fig. 4*D*), survival (Fig. 4*E*), motor coordination, and muscle strength (Fig. 4, *F* and *G*).

Our mouse presents molecular and cellular features very similar to those characterizing the human form of MLII. We next investigated whether it could mimic the behavioral features as well.

##### Progressive Neurodegeneration in the Nymphe Mouse

**FIGURE 5.**
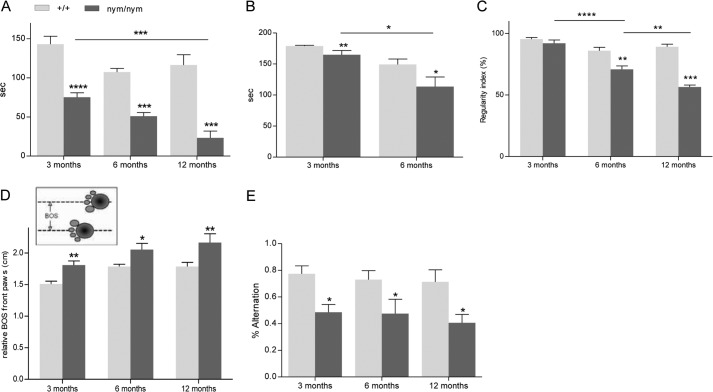
**Impairments in motor and cognitive function.** *nym* (*nym/nym*) mice present with reduced motor coordination on the rotarod (*A*) and reduced muscle strength (*B*), which was measured on the inverted screen. *C*, furthermore, gait analysis was conducted via the Noldus Catwalk system, in which most prominently the regularity index was significantly reduced and progressed with age in the *nym* mouse in comparison with the wild-type (+/+) control. *D*, the regularity index was not significant at 3 months; however, the base of support (*BOS*) for the front paws was significantly increased, and this indicates that the front paws are wider apart than in the wild-type control, giving more stability. *E*, percentage of spontaneous alternation in the Y-maze. Values are expressed as mean ± S.E. (*error bars*) (*n* = 8; *, *p* < 0.05; **, *p* < 0.01; ***, *p* < 0.001).

*nym* mice developed progressive abnormality of gait and showed limb-clasping reflexes from 5 months. Hind limb clasping is an indication of motor imbalance and occurs in various neurodegenerative mouse models (33, 34). Because psychomotor retardation is impaired in MLII patients and is a consequence of slowness in mental activity and locomotion, we first investigated the motor function of the mice using a rotarod and inverted screen to assess motor coordination and strength, respectively. Indeed, the mouse showed reduced muscle strength on the inverted screen test and reduced motor balance and coordination on the rotarod at 3, 6, and 12 months (Fig. 5, *A* and *B*). The rotarod and inverted screen performances also worsened with age, as seen at 6 and 12 months (Fig. 5, *A* and *B*).

Impaired motor strength and coordination was shown with the rotarod and inverted screen; hence, locomotion was subsequently analyzed in more detail by using Noldus Catwalk automated gait analysis (26) to confirm an ataxic gait. A progressive and highly significant reduction of the regularity index, a measure of regular footstep pattern, was observed between 6 and 12 months of age (Fig. 5*C*). Furthermore, the front paw base of support was significantly increased from 3 months of age, indicating reduced stability in the *nym* mice as early as 3 months (Fig. 5*D*).

Because MLII patients have prominent psychomotor retardation with slowness in mental activity, we conducted a preliminary test investigating cognitive deficits in this mouse model. The Y-maze spontaneous alternation task was employed, and it gives a measure of working or short term memory (35). The hippocampus and the frontal cortex have been proposed to be important for these functions (36). In contrast to wild-type mice, which show a high level of performance in this task, *nym* mice just about reach chance levels, which is defined as 50% alternation in choosing an arm at random (Fig. 5*E*). We do not observe a progression of performance with age. These results demonstrate for the first time behavioral deficits in psychomotor performance in a mouse model of MLII, which is a typical finding in patients.

**FIGURE 6.**
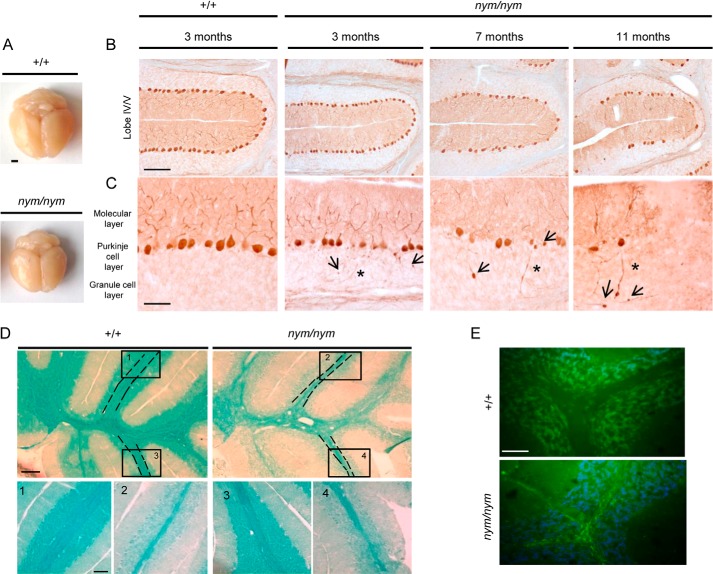
**Pathological alterations in the cerebellum.** *A*, the *nym* (*nym/nym*) brain is about 30% smaller than the wild-type (+/+) brain. *Scale bar*, 3 mm. *B*, parasagittal brain sections were immunostained for calbindin. Analysis of PC degeneration in the whole cerebellum in 3-, 7-, and 11-month-old *nym* mice showed progressive PC loss. Lobe IV/V is shown as a representative lobe of the cerebellum. The majority of Purkinje cells in lobe IV/V are still present at 3 months, but only 75% remain at 7 months, and only 40–50% remain at 11 months. *Scale bar*, 100 μm. *C*, higher magnification images of the 3-month-old *nym* brain revealed that axonal spheroids/torpedoes precede Purkinje cell loss. Swelling of Purkinje cell axons (*arrow*) and neuronal torpedoes (*) in the white matter are observed already at 3 months and increase in severity with age. *Scale bars*, 50 μm. *D*, luxol fast blue staining of the cerebellar sections indicates decreased myelination and degeneration of the subcortical white matter in the *nym* mouse. Higher magnification images are shown below in *panels 1–4* (*1* and *3*, +/+; *2* and *4*, nym/nym). *Scale bars*, 200 μm (*top*) and 100 μm (*bottom*). *E*, strong immunoreactivity for glial fibrillary acidic protein (*GFAP*) in the cerebellum of the *nym* mouse brain. *Scale bar*, 50 μm.

Ataxia typically results from dysfunction of the cerebellum that controls balance and motor coordination; hence, we examined the cerebellum and brain for any gross pathology. The *nym* brain is about 30% smaller than the wild-type brain, including the cerebellum (Fig. 6*A*). We investigated whether the mutant has any defects in the cerebellum structure as well as cell number. Immunohistochemical staining for calbindin showed a substantial loss of Purkinje cells, the main cell output to the cerebellum, in 11-month-old *nym* mice with onset from 7 months (Fig. 6*B*). The Purkinje cell loss and atrophy of brain tissue worsen over time, as seen at 7 months and with about 50% Purkinje cell loss at 11 months (Fig. 6*B*). At 3 months, there is no Purkinje cell loss present; however, we already observe axonal spheroid formation and axonal torpedoes typical of cell atrophy that worsens in severity with age (Fig. 6*C*). Cell death is often accompanied by an inflammatory response and a defect in myelination. Demyelination of the cerebellum occurred with thinning of the white matter tracts (Fig. 6*D*). The nym mice had a reduced level of lipoprotein, which is the binding partner for the luxol fast blue stain. Inflammatory response was present, as seen by an increase in GFAP immunoreactivity throughout the whole brain of *nym* mice, the most affected tissue being the cerebellum. Astrogliosis was present in the white matter and granular and Purkinje cell layer (Fig. 6*E*).

**FIGURE 7.**
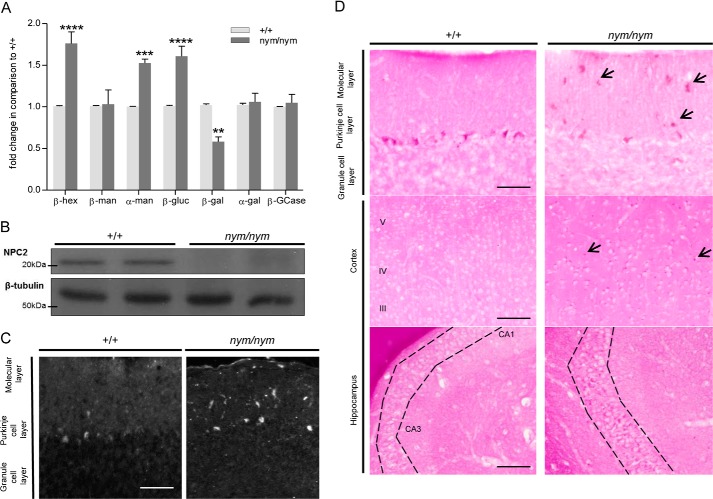
**Biochemical alterations in the brain.** *A*, the relative enzyme activities of the lysosomal hydrolases β-hexosaminidase (β-*Hex*), β-mannosidase (β-*Man*), α-mannosidase (α-*Man*), β-glucuronidase (β-*Glu*), β-galactosidase (β-*Gal*), α-galactosidase (α-*Gal*), and β-glucocerebrosidase (β-*GCase*) were measured in whole brain homogenates of wild-type (+/+) and *nym* (*nym/nym*) mice (4 months of age). The specific activities of the wild type were set to 1. Values are expressed as mean ± S.E. (*error bars*) (*n* = 4; *, *p* < 0.05; **, *p* < 0.01; ***, *p* < 0.001; ****, *p* < 0.0001). *B*, representative Western blot showing levels of Npc2 in wild-type and *nym* mice. β-Tubulin was used as a loading control (*n* = 4). *C*, nine-month-old *nym* and wild-type brain sections were stained with filipin for the detection of cholesterol. The molecular layer of the *nym* cerebellum showed increased storage in comparison with the wild-type control. *Scale bar*, 50 μm. *D*, nine-month-old *nym* and wild-type brain sections were stained with periodic acid-Schiff for detection of glycolipids. Increased storage was detected in the cerebellum (*top*, specifically in the molecular layer), the cortex (*middle*, specifically in layers 3–5), and the hippocampus (*bottom*, specifically in layers CA1 and CA3). *Scale bar*, 50 μm.

The activity of lysosomal enzymes was also deregulated in brain homogenates from the *nym* mice. β-Hexosaminidase, α-mannosidase, and β-glucoronidase were increased by 1.5-fold, whereas β-galactosidase was significantly decreased by 30% (Fig. 7*A*). Lysosomal enzyme activities for brain homogenate in nmol/mg/h can be seen in Table 2. Furthermore, we found a loss of expression of the Npc2 protein in the *nym* brain (Fig. 7*B*). Due to this deregulation of lysosomal enzymes, it was of interest to analyze which substrates accumulate in the *nym* brain. Periodic acid-Schiff-stained brain sections revealed increased glycolipid and cholesterol storage in the cerebellum, cortex, and hippocampus (Fig. 7, *C* and *D*). Specifically, the cerebellar molecular layer had increased storage of glycolipids in comparison with the granular layer (Fig. 7*D*, *top*). Large amounts of storage of glycolipids were present in the cortical layers 3–5 and lesser amounts in layers 1 and 2 (Fig. 7*D*, *middle*). Furthermore, the hippocampal layers CA1 and CA3 presented with increased glycolipid storage (Fig. 7*D*, *bottom*). These findings agree with what is observed in NPC mouse models and α-mannosidosis causing GM2 accumulation (37, 38).

**TABLE 2:** **Lysosomal enzyme activities (nmol/mg/h) in brain lysate of wild-type and *nym* (*nym/nym*) mouse**

Lysosomal enzyme	Wild type (+/+)	*nym/nym*
β-Hexosaminidase (nmol/mg/h)	1.39 ± 0.19	2.24 ± 0.22
β-Mannosidase (nmol/mg/h)	0.71 ± 0.09	0.70 ± 0.09
α-Mannosidase (nmol/mg/h)	0.47 ± 0.07	0.70 ± 0.02
β-Glucoronidase (nmol/mg/h)	0.54 ± 0.07	0.87 ± 0.05
β-Galactosidase (nmol/mg/h)	1.60 ± 0.24	0.99 ± 0.10
α-Galactosidase (pmol/mg/h)	42.42 ± 11.57	45.34 ± 3.41
Glucocerebrosidase (pmol/mg/h)	16.70 ± 3.96	16.98 ± 5.97

**FIGURE 8.**
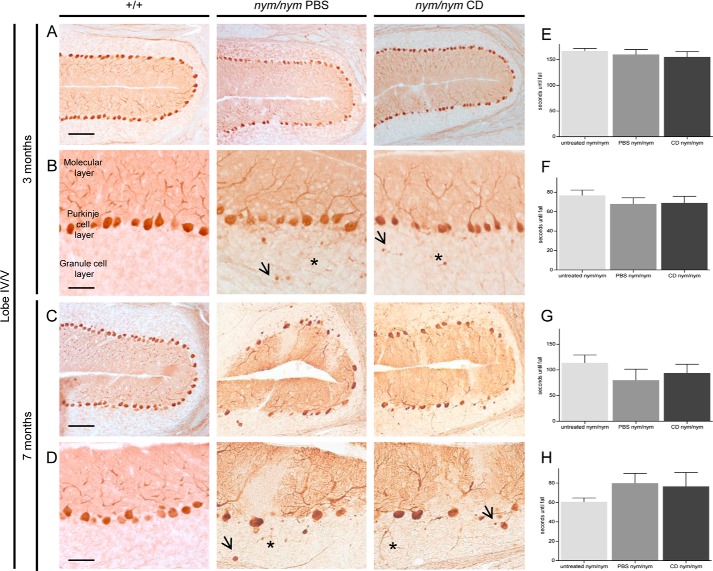
**Cyclodextrin does not delay cerebellar pathology and motor impairments.** P7 *nym* (*nym/nym*) mice were injected weekly for 7 months with cyclodextrin (*CD*) at 4000 mg/kg or the vehicle PBS (*n* = 7 in each group). *A*, no PC loss was observed at 3 months, as can be seen in lobe IV/V as a representative lobe of the cerebellum. *Scale bar*, 100 μm. *B*, typical signs of PC degeneration, including swelling of PC dendrites (*arrow*) and axonal torpedoes (*), can be observed. *Scale bars*, 50 μm. *C*, PC loss was observed at 7 months in lobe IV/V as a representation of a lobe of the cerebellum in *nym* PBS- and *nym* CD-treated mice. *Scale bar*, 100 μm. *D*, typical signs of PC degeneration, including swelling of PC dendrites (*arrow*) and axonal torpedoes (*), can be observed. *Scale bars*, 50 μm. *E–H*, 3- and 7-month-old cyclodextrin (*CD*) treated *nym* mice perform the same as PBS and untreated *nym* mice on the inverted screen (3 months (*E*) and 7 months (*G*)) and rotarod (3 months (*F*) and 7 months (*H*)). Values are expressed as mean ± S.E. (*error bars*) (*n* = 8). +/+, wild-type mice.

Interestingly, the *Npc2* disease mouse model shows a brain pathology very similar to that of the *nym* mouse with Purkinje cell degeneration, suggesting that loss of Npc2 function may be a factor driving this aspect of pathogenesis (39, 40). In the NPC disease model, the drug 2-hydroxypropyl-β-cyclodextrin has been reported to delay Purkinje cell loss and is currently in clinical trials for patients with this disorder (41, 42). To test whether dysfunction of the Npc2 pathway is also implicated in the brain pathogenesis of the *nym* mouse, we treated this mutant with 2-hydroxypropyl-β-cyclodextrin for 7 months, when we started seeing Purkinje cell loss. However, we did not see any rescue or delay in the cerebellar pathology at 3 months or at 7 months (Fig. 8, *A–D*), and neither motor improvement was observed at both of these ages (Fig. 8, *E–H*). These findings indicate that the loss of Npc2 function is not the primary pathogenic mechanism that triggers Purkinje cell degeneration in MLII disease.

## DISCUSSION

In this study, we describe a novel mouse model of MLII, which carries a premature stop codon mutation previously reported in a patient (12). Importantly, unlike the existing gene knock-out mouse model, it more completely recapitulates the characteristic pathological features observed in patients, including facial and skeletal abnormalities, mislocalization of lysosomal enzymes, abnormal intracellular storage, and also psychomotor retardation, motor dysfunction, and reduced life span (31).

The *nym* mouse resembles closely a recently described knock-in mouse of another MLII human mutation, the G1028X mouse equivalent (43). Both mutations lead to premature stop codons. The *nym* mutation Y867X leads to the production of a slightly truncated α-subunit retaining 95% of the wild-type equivalent and a complete lack of β-subunit, and the knock-in mutation G1028X carries a complete α- and a truncated β-subunit (Fig. 1*B*). Both mutations lead to a loss of the C terminus of the β-subunit that contains a transmembrane domain and ER exit motif (R/K)*X*(R/K). Without the ER exit motif, the protein will reside in the ER and not locate to the Golgi (Fig. 1*D*). This will ultimately lead to a non-functional Gnpta protein because the assembly of α- and β-subunit will not occur because the cleavage of the precursor protein occurs at the Golgi (15), to which the mutant protein will not locate.

Both mutants show several biochemical and clinical features of the disease MLII, including skeletal abnormalities and facial dysmorphisms. A phenotypic comparison of the different MLII mouse models is summarized in Table 3. The *nym* spine presents with a thoracic curve in contrast to the wild-type and the knock-in (Table 3) (44). This might be because the knock-in was analyzed at 4 weeks in comparison with 12 months for the *nym* mouse, in which the phenotype might have progressed. Both mice present with reduced life span; however, the *nym* mouse presents with a survival of ∼30% at 60 weeks, whereas the knock-in presents with a 2% survival chance at 60 weeks. We are uncertain about the reason, but it might be because of difference in background strain, husbandry effects, or *n*-numbers. Only 33 *nym* mice were analyzed in terms of survival in comparison with 160 animals for the knock-in (43).

**TABLE 3:** **Comparison of the *nym* mouse with *Gnptab* knock-in and knock-out mouse**

MLII model	Murine *nym* mutant	Murine knock-out	Murine knock-in
Mutation	Patient mutation (c.2664C>G) in mouse: c.2601T>A leading to a truncation mutation	*Gnptab* Gene TRAP mouse (22)	Patient mutation (c.3145insC) leading to a protein truncation G1028X (43)
Phenotype	Reduced life span	Normal life span	Reduced life span
	Growth retardation	Growth retardation	Growth retardation
	Skeletal abnormalities	No skeletal abnormalities	Skeletal abnormalities
	Craniofacial defects	No craniofacial defects	Craniofacial defects
	Ataxic gait and reduced muscle strength and motor coordination analyzed by catwalk, rotarod, and inverted screen	No report of ataxic gate	Presented with ataxic gait
	Mental retardation shown by the spontaneous alternation task	No report of behavioral dullness	No report of behavioral dullness
	Inclusion bodies present in fibroblasts	Inclusion bodies present in secretory organs (pancreas) and connective tissue (cartilage)	Inclusion bodies present in fibroblasts
	Not analyzed	Retinal impairments	Retinal impairments

The neurodegenerative phenotype is very similar between the knock-in and the *nym* mouse, both presenting with cerebellar pathology, including progressive Purkinje cell loss, demyelination, inflammation. Due to the lysosomal enzymes not being localized to the lysosomes correctly, these are unable to break down waste material, and storage of gangliosides and cholesterol occurs in a variety of different brain regions. These have not been reported for the knock-out. Also, the *Gnptab* knock-out mouse does not display all of the clinical features and symptoms of MLII patients that are seen in the cat MLII model, knock-in, and *nym* mice (Table 3) (22, 23). The *Gnptab* knock-out mice have normal presentation of liver, brain, and muscle tissue. However, immunohistochemical analysis of brain sections revealed the presence of age-dependent lesions and reactive microgliosis, and they presented with hind limb-clasping at 4–6 months of age (43). Hence, the *Gnptab* knock-out brain represents a less severe course of disease than observed in *nym* or knock-in mice. Lysosomal enzyme activities in serum show very similar trends in *nym* and knock-in mice but also in the knock-out mouse despite the difference in phenotype. Therefore, missorting of lysosomal enzymes is most probably not causing the difference in phenotype but potentially the residual protein of Gnpta in knock-in and *nym* mice that exerts a potential toxic gain of function. Our results highlight the value of clinically relevant point mutants compared with null models because the null does not present with patient relevant pathology of reduced life span, neurodegeneration, and skeletal and facial abnormalities (Table 3).

Psychomotor retardation is a common pathology in MLII patients; however, neurodegeneration has so far not been reported in MLII patients, except for minimal signs of neuronal and glial involvement (45). Patients die in their first decade of life, in which we would expect Purkinje cell death and early markers of neurodegeneration (*e.g.* inflammation, neuronal torpedoes, and swelling of Purkinje cell axons with formation of spheroids in the granular layer) to be present. No investigations have been conducted so far in MLII patients, but the early markers might be cause for psychomotor retardation and should be investigated further.

A well established link between cerebellar pathology and motor dysfunction has already been made (46) and is supported by many cerebellar mutant mice that have ataxic phenotypes, namely *Lurcher*, *hot-foot*, and *staggerer* (47). In the past decade, the cerebellum has also emerged as an important brain region for the control of higher cognitive functions (48). Connections run from the prefrontal cortex to the cerebellum that confirm involvement in cognitive networks (49). Indeed, reports of cerebellar ataxia and psychomotor retardation segregate in many studies, such as in Cayman cerebellar ataxia (50), isolated cerebellar hypoplasia (51), and lysosomal storage disorders, such as Niemann-Pick type C disease (52). The spontaneous alternation task, which we conducted on the *nym* mice, is a measure of working or short term memory (35), and the hippocampus and the frontal cortex have been argued to be important for these functions (36). Microglial activation was observed in the knock-in mouse in all brain regions. The strongest intensity was observed in the cerebellum, cerebral cortex, hippocampus, and thalamus (43). This could explain why the *nym* mouse underperformed in the spontaneous alternation task. This is the first behavioral observation that these mice not only have motor slowness assessed by rotarod, catwalk, and inverted screen but also cognitive slowness challenged in the alternation task. Behavioral dullness is a hallmark of the human pathology and has been observed in the feline model of MLII but so far has been observed in no other mouse model, which supports the *nym* mouse being a human disease-relevant model (12, 18). Further research will be necessary to confirm the exact brain region of dysfunction via measuring electrical activity in different brain regions to observe abnormalities in long term potentiation, which is known to be involved in memory formation (53).

Targeting efficiency of lysosomal enzymes is cell type- and tissue-specific in human and disease models of MLII (8, 54–56). Storage of glycolipids was observed in certain cell types of the *nym* brain. The M6P pathway is important in targeting the majority of lysosomal hydrolases to the lysosome, so high levels of storage would be anticipated. However, the storage of glycolipids and the pathology in general is not as severe as in single enzyme deficiency models, such as Sandhoff disease, in which greater glycolipid storage is observed and the mice have a greatly attenuated life span (57, 58). This highlights the importance of alternative pathways that can deliver lysosomal hydrolases to the lysosomes and need to be further studied, which is in agreement with the observations of Braulke and co-workers (8, 43). Here, we showed that the loss of mannose 6-phosphate residues in *nym* mice led to a loss of the Npc2 protein that is involved in the lysosomal export of cholesterol and sphingolipids in the *nym* brain. Due to the missorting of Npc2, we conducted a drug trial over 7 months, the age when we observe Purkinje cell death, with 2-hydroxypropyl-β-cyclodextrin. This drug has shown to increase the life span and delay Purkinje cell loss in Npc2 knock-out mice (41). However, this treatment did not delay Purkinje cell loss or motor impairment, suggesting that the reduced levels of lysosomal Npc2 in MLII is not the primary mechanism that triggers Purkinje cell degeneration. Taken together, our findings in this novel mouse model of MLII suggest that it will be a useful system to better understand pathogenic mechanisms and evaluate modifying therapies of disease.

## Footnotes

5 The abbreviations used are:

MLII: mucolipidosis II
NPC: Niemann-Pick type C
M6P: mannose 6-phosphate
GNPTA: GlcNAc-1-phosphotransferase
MEF: mouse embryonic fibroblast
ER: endoplasmic reticulum
GM2: monosialoganglioside 2.
